# Droplet digital PCR as a tool for investigating dynamics of cryptic symbionts

**DOI:** 10.1002/ece3.8372

**Published:** 2021-11-22

**Authors:** Anna‐Lotta Hiillos, Anne Thonig, Karelyn Emily Knott

**Affiliations:** ^1^ Department of Biological and Environmental Science University of Jyväskylä Jyväskylä Finland; ^2^ Department of Science and Environment Roskilde University Roskilde Denmark

**Keywords:** apicomplexa, cryptic symbiosis, droplet digital PCR, infection dynamics

## Abstract

Interactions among symbiotic organisms and their hosts are major drivers of ecological and evolutionary processes. Monitoring the infection patterns among natural populations and identifying factors affecting these interactions are critical for understanding symbiont–host relationships. However, many of these interactions remain understudied since the knowledge about the symbiont species is lacking, which hinders the development of appropriate tools. In this study, we developed a digital droplet PCR (ddPCR) assay based on apicomplexan COX1 gene to detect an undescribed agamococcidian symbiont. We show that the method gives precise and reproducible results and enables detecting cryptic symbionts in low target concentration. We further exemplify the assay's use to survey seasonally sampled natural host (*Pygospio elegans*) populations for symbiont infection dynamics. We found that symbiont prevalence differs spatially but does not show seasonal changes. Infection load differed between populations and was low in spring and significantly increased towards fall in all populations. We also found that the symbiont prevalence is affected by host length and population density. Larger hosts were more likely to be infected, and high host densities were found to have a lower probability of infection. The observed variations could be due to characteristics of both symbiont and host biology, especially the seasonal variation in encounter rates. Our findings show that the developed ddPCR assay is a robust tool for detecting undescribed symbionts that are otherwise difficult to quantify, enabling further insight into the impact cryptic symbionts have on their hosts.

## Graphical abstract

Many symbiont‐host interactions are understudied due to the lack of knowledge about the symbiont species hindering the development of appropriate tools. Here, we developed a ddPCR assay to detect an undescribed agamococcidian symbiont in its host, *Pygospio elegans*, and show that the method enables detecting cryptic symbionts even in low target concentration. We further exemplify the assay's use to survey seasonally sampled natural host populations for symbiont infection dynamics.

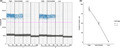

## 1 INTRODUCTION

Interactions between symbiotic organisms and their hosts dramatically influence not only organismal ecology and evolution, but also the dynamics of entire ecosystems (Faust & Raes, 2012; Godfrey‐Smith, 2015). For the majority of symbioses, however, we do not know how the interacting species affect each other, much less the broader consequences of the symbiosis for communities or ecosystems. Often, it is the lack of tools for effectively monitoring or investigating symbiotic interactions that prohibits progress. In addition, a lack of general knowledge about the symbiotic organisms can limit the development of these tools (Pawlowski et al., 2012), leaving much of the functional and species‐level diversity understudied.

Apicomplexans are a diverse group of microbial eukaryotes that include some of the most studied parasites with significant social and economic consequences (e.g., the genera *Plasmodium*, *Toxoplasma*, *Eimeria*, and *Cryptosporidium*) (Seeber & Steinfelder, 2016). Infecting majority of vertebrates and invertebrates in terrestrial and aquatic environments, apicomplexan species diversity is estimated to be over a million (Pawlowski et al., 2012). However, the bulk of apicomplexan diversity, especially in marine environments, is largely undescribed and contains many cryptic species (Janouškovec et al., 2015; Xavier et al., 2018). Additionally, despite the fact that some groups are known to be common and prevalent symbionts of marine invertebrates, little is known about the nature of their interactions (mutualistic or parasitic) with their hosts (Rueckert et al., 2019). Studying symbiont dynamics, even when the symbiont is cryptic, could help to shed light on the nature of their interaction with the host and their effect on host populations.

The presence of apicomplexans within host individuals and populations is traditionally determined with microscopy, but this can be difficult and labor intensive when studying large samples and may overlook small or cryptic microbial eukaryotes. In such cases, the use of molecular tools, such as PCR methods or amplicon sequencing, often are more appropriate. For instance, real‐time quantitative PCR (qPCR) has been widely used in detecting and quantifying protistan parasites (e.g., Maia et al., 2014). Digital PCR (dPCR) is a third generation PCR method for quantification of target molecules in a nucleic acid sample (Li et al., 2018). It is based on partitioning and randomly distributing the sample into small partitions before PCR amplification, which takes place in each partition separately (Hindson et al., 2011). After amplification, the end‐point reaction is visualized to determine the fraction of positive partitions (Hindson et al., 2011, 2013). From this fraction, the concentration of the target can be estimated using Poisson statistics (Sykeset al., 1992). In droplet digital PCR (ddPCR), the sample is partitioned into up to 20,000‐nl‐sized droplets by water–oil emulsion and the target concentration is therefore determined from the fraction of positive droplets. The advantage of partitioning lies in increased resolution and sensitivity; hence, ddPCR applications have been widely used in clinical studies, GMO detection, and food security (Demeke & Dobnik, 2018; McMahon et al., 2017; Sedlak et al., 2014).

Compared with traditional real‐time qPCR, ddPCR has several major advantages. For example, it has higher precision and day‐to‐day reproducibility, especially when targeting rare copies (Brys et al., 2020; Doi et al., 2015; Hindson et al., 2013). ddPCR assays also tolerate inhibition better than qPCR (Dingle et al., 2013; Poh et al., 2020; Rački et al., 2014), and the end‐point measurement of the nucleic acid quantitation is not affected by amplification efficiency. In addition, ddPCR does not require standard curves (Volgenstein & Kinzler, 1999). Therefore, the concentration of the target copies is an absolute measurement that is not reliant on Cq values. Furthermore, there is increased statistical power of ddPCR over qPCR (Taylor et al., 2017). These advantages make the method particularly applicable for molecular identification of symbionts. The increase of studies using ddPCR for quantifying haemoprotozoan infections (e.g., *Plasmodium* and *Babesia*) (Koepfli et al., 2016; Srisutham et al., 2017; Wilsonet al., 2015) and for detecting parasites from environmental samples (Mulero et al., 2020; Rusch et al., 2018) has already shown that the method is robust, reproducible, and capable of detecting rare copies of target DNA.

Despite the aforementioned advantages, ddPCR has not been used to its full potential, in particular for investigating cryptic symbionts and their ecological interactions with hosts. In this study, we show that ddPCR can shed light on biological interactions with precise estimates of cryptic, unculturable symbionts in natural host populations, indicating that the method is useful in molecular ecology research. We introduce a detailed ddPCR protocol and demonstrate its use by investigating the seasonal dynamics of an undescribed cryptic agamococcidian (Apicomplexa) infection for the first time in four polychaete (*Pygospio elegans*; Claparède, 1863) populations. By using specific primers, our assay targets the mitochondrial cytochrome oxidase *c* subunit 1 gene (COX1) of the agamococcidian symbiont and can be used effectively to determine symbiont prevalence and infection load from whole host DNA extracts.

## 2 MATERIALS AND METHODS

### 2.1 Study organisms

The host species, *P. elegans*, is a small polychaete worm that inhabits sandy coastal habitats throughout the northern hemisphere. This species can be a dominant member of benthic communities (Bolam, 2004; Bolam & Fernandes, 2003) and is an important prey item for other invertebrates and fish (Mattila, 1997). *Pygospio elegans* is known to host at least two apicomplexan symbionts: the archigregarine *Selenidium pygospionis* (Paskerova et al., 2018) and the eugregarine *Polyrhabdina pygospionis* (Paskerova et al., 2021), which both inhabit the worm's intestine. In our previous study of the host's transcriptome (Heikkinen et al., 2017), we detected the presence of a third apicomplexan symbiont. Based on 18S rDNA sequence similarity, this symbiont is most likely an agamococcidian (Order Agamococcidiorida; Levine, 1976), but its location within the host and definitive identification is not yet known. Agamococcidians are a small group of coccidian‐like symbionts currently comprising two monogeneric families Gemmocystidae and Rhytidocystidae (Levine, 1976; Upton & Peters, 1986), but the taxonomy of the group has been questioned (Janouškovec et al., 2019; Mathur et al., 2020). Rhytidocystidae includes species infecting midguts of marine polychaetes (Leander & Ramey, 2006; Miroliubova et al., 2020; Rueckert & Leander, 2009), and some have been suggested to have intracellular life stages at least at an early stage of development within the hosts (Miroliubova et al., 2020). Because research on this group has mostly focused on resolving their phylogenetic position and describing the species, the nature of their interaction with their hosts has not been studied previously.

### 2.2 ddPCR assay specificity

Our goal was to produce an assay that could be used to detect and estimate infection loads of a cryptic symbiont from total DNA extracts (containing a mixture of host and symbiont DNA). A limited dataset of potential genes was available from our previous transcriptome study of the host (Heikkinen et al., 2017). We chose to design primers to amplify the presumed mitochondrial COX1 gene of the undescribed agamococcidian, as it showed sufficient divergence from the host's COX1 sequence. Primer3 software (https://primer3.ut.ee) was used to choose appropriate primers: forward primer ApiCox1F (5′‐ACT GGT CTA TCA AGT GTA CTG GC‐3′) and reverse primer ApiCox1R (5′‐GAT CAC CAC TAA ATT CAG GGT CA‐3′) to amplify 226 bp of the target gene. Amplicons were identical in sequence to the previously obtained transcript and showed high similarity to transcripts obtained from *Rhytidocystis* sp. ex. *Travisia forbesii* (Supporting information 1). Amplification efficiency of the primers was estimated to be 94.3% (slope = −3.465, *r* ^2^ = .997) using a qPCR. To demonstrate the specificity of the designed primers, the assay was checked against other apicomplexan DNA that might be present in the host gut; *S. pygospionis* (Archigregarinorida) (Paskerova et al., 2018) and *P. pygospionis* (Eugregarinorida) (Paskerova et al., 2021). Individual *S. pygospionis* (*n* = 88) and *P. pygospionis* (*n* = 33) cells were isolated from *P. elegans* collected in September 2020 (St. Petersburg, Russia). Non‐attached gregarines were hand‐picked from dissected host intestines by micromanipulation and washed three times with filtered (Millipore 0.2 µm) seawater. The cells were then transferred into microtubes, pelleted down with centrifugation to remove excess water, and stored in 100% EtOH for transport to our laboratory. Prior to DNA extraction, EtOH was evaporated from the samples and the DNA was extracted using MasterPure™ Complete DNA and RNA Purification Kit (Lucigen) following the manufacturer's protocol, except for the elution step, which was done in 10 μl of EB buffer. DNA concentration was checked using a Qubit 4.0 Fluorometer with 1X dsDNA HS Assay (Thermo Fisher Scientific).

### 2.3 ddPCR protocol: reproducibility, dynamic range, and the limit of detection

ddPCR was performed using Bio‐Rad's QX200™ Droplet Digital™ PCR System. Reproducibility of the assay was inspected with four replicates of three DNA samples: host with high (approx. 100 copies/µl), intermediate (approx. 50 copies/µl), and low (approx. 2 copies/µl) infection load. Because samples are partitioned into droplets in sets of eight in the QX200 system, two replicates of each sample were used in two different droplet generation events (four replicates for each sample). To inspect the dynamic range of the assay and the limit of detection (LOD), a 10‐fold dilution series (1, 1:10, 1:100, and 1:1000) of the DNA extracted from the host with high infection load was performed in 10 replicates. Linearity over the dynamic range was determined by the coefficient of correlation *r* ^2^, calculated on the target concentration (copies/µl) measured in the dilution series replicates. Assay repeatability was determined by the % coefficient of variation (%CV = concentration standard deviation/concentration mean * 100) between the replicates. LOD is defined as the lowest target copy number in a sample that can reliably be detected. In this study, LOD was determined as the lowest concentration level for which all 10 replicates resulted in at least three positive droplets per replicate. In all experiments, a negative control containing nucleotide free water was used. The reaction mix was prepared to a volume of 20 µl per sample. We used 2X QX200™ ddPCR™ EvaGreen^®^ (Bio‐Rad) reagent mix. Primers were added to the mix in 1 µM together with 4.6 µl of sterile water. The reaction mix was divided into individual 0.5 ml microfuge tubes, and 2 µl of the DNA templates (varying concentration) was added to each tube so that the final reaction volume was 22 µl. Samples were partitioned into nanoliter‐sized droplets with the QX200 Droplet Generator (Bio‐Rad) using single‐use DG8 cartridges and Droplet Generation Oil (Bio‐Rad). Twenty microliters of each reaction mix was loaded to the cartridge, and the emulsion was made with 70 µl of oil. The resulting droplets were manually transferred with a multichannel pipet to a ddPCR™ 96‐well PCR plate (Bio‐Rad), which was heat‐sealed with a foil cover.

The droplets were then subjected to thermocycling using a Bio‐Rad C1000 thermocycler with a ramp rate of 2°C/s in each step. Initial denaturation of the DNA was done at 95°C for 3 min, after which the denaturation, primer annealing, and target extension steps were repeated for 40 cycles. The denaturation step was done at 95°C for 30 s, annealing temperature for the primers was optimized at 58°C for 1 min, and the target extension step was done at 72°C for 2 min. After the cycles, a signal stabilization step from 5 min at 4°C to 5 min at 90°C was added. Following the amplification, the droplets were immediately read with Bio‐Rad's Droplet Reader.

### 2.4 ddPCR data analysis

Absolute quantification of target gene copies was done with default ABS settings in QuantaSoft Analysis Pro 2.0 software (Bio‐Rad). The ABS experiment estimates the concentration of the target in copies per microliter of the final 1X ddPCR reaction. Because one droplet can harbor one or more copies of the target, or none, the target concentration (copies/µl) is estimated by the software by calculating the mean copies per partition (*λ*) following Equation (1), where *n* is the total number of accepted droplets and *k* is the number of positive droplets counted.

(1) $$\lambda =‐\ln\left(1‐\frac{k}{n}\right)$$

The infection load (copies/ng total DNA) was calculated using the reaction mix volume (22 µl), the sample volume (2 µl), and the concentration (ng/µl) of the DNA sample following Equation (2), where *C*_ng_ is the number of copies per nanogram of total DNA, *C*_ddPCR_ is the reaction concentration (copies/µl) given by QuantaSoft Analysis Pro, *V*_r_ is the reaction mix volume, *V*_s_ is the sample volume, and *C*_DNA_ is the concentration (ng/µl) of total DNA sample.

(2) $$C_{\text{ng}}=\left(\frac{C_{\text{ddPCR}}\times V_{r\;}\left(\mu l\right)}{V_{s}\left(\mu l\right)}\right)/C_{\text{DNA}}$$

Only reactions that had ≥10,000 droplets were included in further analyses. A threshold to separate the target positive and negative droplets was manually set in relation to the negative control by visual inspection.

### 2.5 Survey of host populations

**FIGURE 1:**
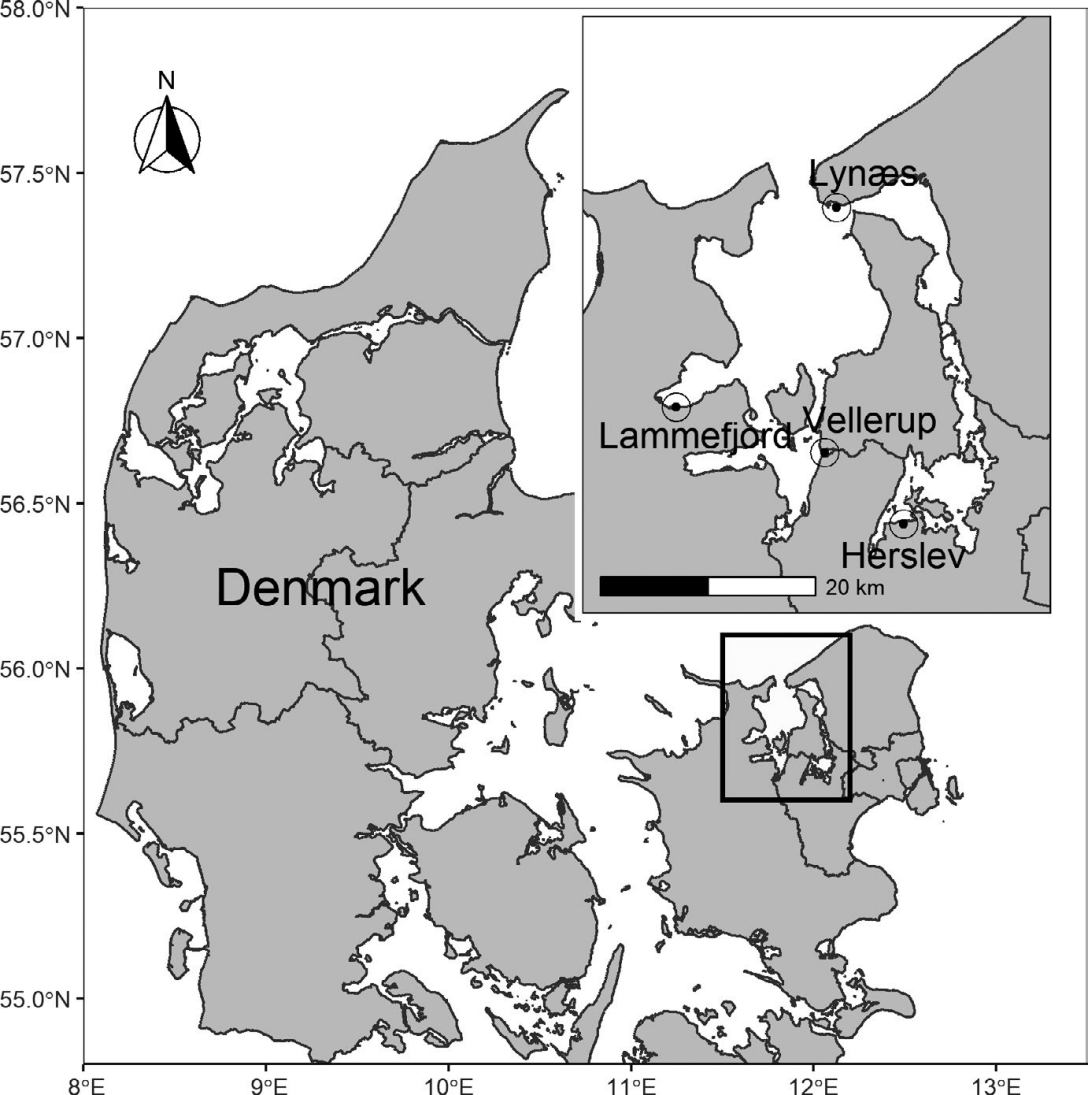
Location of the four sampling sites in Isefjord‐Roskilde Fjord complex, Denmark

To determine the dynamics of the symbiotic infection in natural host populations, we quantified the agamococcidian COX1 gene copies in DNA extracts from whole *P. elegans* individuals collected from four populations in the Isefjord‐Roskilde Fjord estuary at Lynæs, Lammefjord, Vellerup, and Herslev, Denmark (Figure 1). The populations were sampled seasonally in a previous study from March 2014 to February 2015 to describe the reproductive dynamics of the host (see Thonig et al., 2016, for sampling details). In addition, the authors determined size (length in micrometer from eyespot to the beginning of gills) for each host and population density (individuals in square meter). Environmental parameters (salinity, temperature, and sediment characteristics) were also collected seasonally (see Thonig et al., 2016).

DNA was extracted from whole host individuals using DNeasy Blood and Tissue Kit (Qiagen) following the manufacturer's protocol for animal tissue and stored in −20°C (Thonig et al., 2017), and DNA concentration was measured with Qubit 4.0 Fluorometer with 1X dsDNA HS Assay (Thermo Fisher Scientific). In the current study, a subset of those samples was analyzed with ddPCR (Table 1). These sampling times were chosen for quantification of agamococcidian infection due to population specific changes in host reproduction and density patterns observed in these months (Thonig et al., 2016).

**TABLE 1:** Number of host individuals in each population and month studied for agamococcidian infection dynamics

	Herslev	Lammefjord	Vellerup	Lynæs
March	27	25	26	25
May	28	25	27	25
August	25	25	25	25
October	23	25	26	25
November	28	24	22	19

We used the ddPCR protocol described earlier to detect the cryptic symbiont (prevalence) and estimate its abundance (infection load). Prevalence of the symbiont in the host population was measured as the proportion of infected *P. elegans* individuals in the sampled population. An individual host was considered infected if more than 0 copies of the target gene per ng of total DNA were detected with the ddPCR assay. Infection load was defined as the number of COX1 gene copies/ng of total DNA, as in Equation (2), and analyzed with only infected hosts. Aggregation of the infection was inspected by calculating variance to mean ratios.

### 2.6 Statistical analyses

In the ddPCR assay reproducibility experiment, the normality of the data was checked with Shapiro–Wilk test. The concentration did not follow a normal distribution; hence, a non‐parametric Wilcoxon rank sum test was used to compare the replicates of high, intermediate, and low infection load medians between the two droplet generation events. The linearity of dynamic range was determined using least squares regression analysis of the linear relationship between sample quantity (fold) and COX1 concentration (copies/µl) in each dilution series replicates. The correlation of droplet count and the absolute concentration was analyzed for all replicates used in the two experiments described earlier (*n* = 51) with Pearson's correlation.

To avoid multicollinearity issues caused by significant correlations between predictor variables in the infection dynamics analysis, the correlations between the environmental variables and host density were inspected with Pearson's correlation. Organic content % in the sediment was chosen for further analysis, and all other environmental variables were excluded because of high correlations (Table 2). The prevalence of infection in the different populations and months was analyzed by logistic regression using population, sampling month, host length (in micrometer), host density (individuals in square meter), and the organic content % in the sediment as predictors. The log‐transformed infection load was analyzed using linear regression with the same predictor variables. As the host density and the environmental variables were not measured in October, and two hosts did not have the length measurement, the missing values were imputed using Multivariate Imputation with Chained Equations method (mice) (van Buuren & Groothuis‐Oudshoorn, 2011). In total for 99 out of 499 hosts (19.8%), there was no density or organic content measurements in October. We used multiple imputation to create and analyze five multiply imputed datasets. Incomplete variables (length and organic content) were imputed under fully conditional specification, using the predictive mean matching method and 50 iterations per imputation. The parameters were estimated in each imputed dataset separately and combined using Rubin's rules (Rubin, 1987). Each imputation was inspected visually by comparing the original data and the imputed values in a strip plot, and convergence of the iterations was inspected using Markov Chain Monte Carlo typed algorithm. The missing host density values were imputed for each population with linear estimation using the observed densities before (August) and after (November) the missing cases in October. The best fitting model was chosen with likelihood‐ratio test (D3 in mice package) for logistic regression and with Wald test (D1mice package) for linear regression. For comparison, we also performed the analysis using only the samples with complete environmental data (excluding samples from October). All statistical analyses were performed in RStudio version 3.6.1 (05/07/2019) (https://cran.rproject.org).

**TABLE 2:** Pearson correlation coefficients (below diagonal) and the significance of the correlations (above diagonal) for environmental variables and host density

	Density	Mean salinity	Mean T	Mean C/N	Organic content	Water content	Porosity	Sorting *φ*	Median *φ*
Density	—	***	**	***	0.290	***	***	0.470	***
Mean Salinity	−0.25	—	***	***	***	0.079	***	***	0.096
Mean T	0.15	−0.2	—	***	0.077	***	***	*	***
Mean C/N	−0.24	0.19	−0.43	—	***	***	***	***	0.801
Organic content	0.05	0.4	0.09	−0.27	—	***	***	**	***
Water content	−0.39	0.09	−0.26	0.26	0.2	—	***	***	0.841
Porosity	−0.36	0.28	−0.19	0.27	0.38	0.9	—	***	0.297
Sorting *φ*	0.04	0.21	0.1	0.48	−0.15	−0.55	−0.46	—	0.822
Median *φ*	−0.22	−0.09	0.21	0.01	−0.29	−0.01	0.06	0.01	—

Note

Signif. *p < .05. **p < .01. ***p < .001.

## 3 RESULTS

### 3.1 Specificity, reproducibility, and detection limit of the ddPCR assay

**FIGURE 2:**
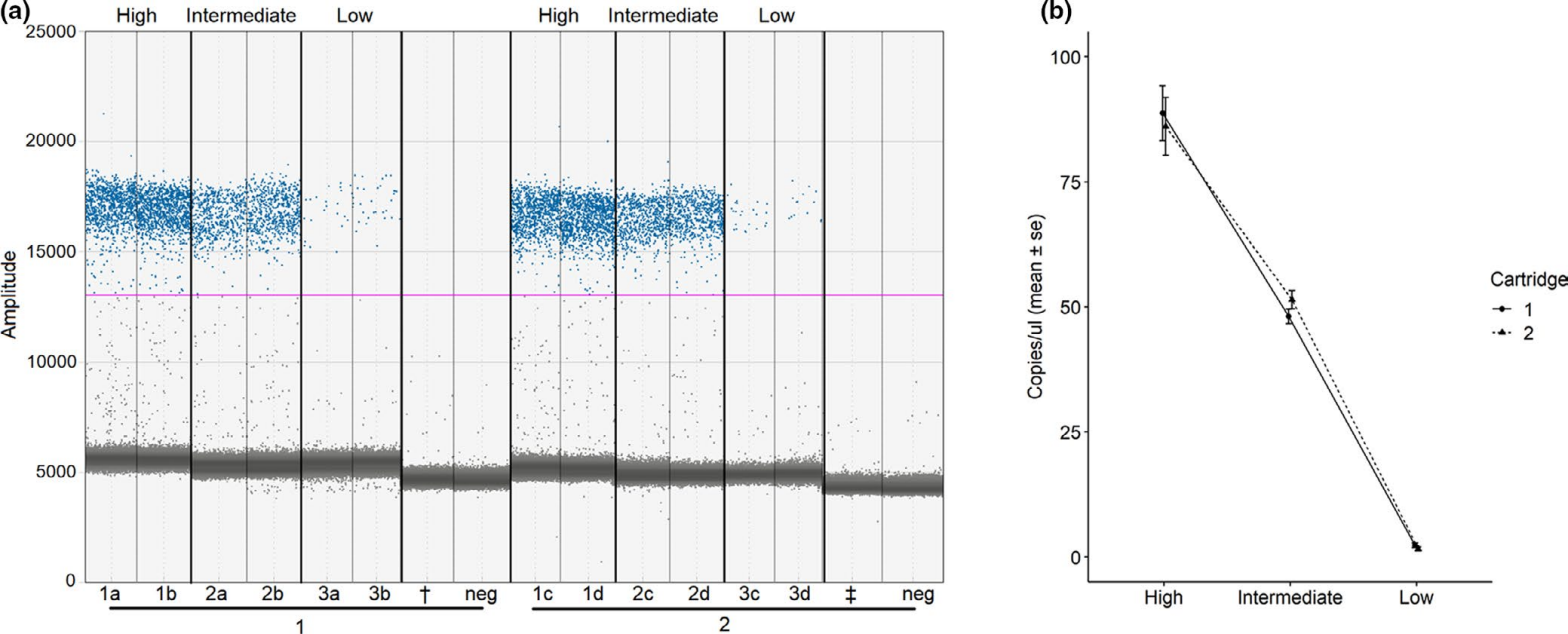
The ddPCR assay for hosts with high, intermediate, and low infection load of the agamococcidian symbiont. (a) 1D ddPCR plot. Replicates a and b contain droplets generated in cartridge 1, and replicates c and d contain droplets generated in cartridge 2. Individual reactions that are shown in the *x*‐axis and *y*‐axis show the amplitude of the fluorescence. Positive reactions (blue) and negative reactions (grey) are separated by a manually set threshold (pink line, at amplitude 13,033). Negative controls (neg) did not show any amplification above the threshold (concentration 0 copies/µl). The other apicomplexans possibly present in the mixed DNA samples (**^†^** *Selenidium pygospionis* and **^‡^** *Polyrhabdina pygospionis*) also showed no amplification. (b) Concentration of agamococcidian COX1 gene copies per µl (mean copies/µl ± SE) in the two droplet generation events, cartridge 1 shown in solid line and black circle, and cartridge 2 shown in dashed line and black triangle

Our ddPCR assay revealed on average 87.8 copies/µl (SE =3.35) of agamococcidian COX1 gene for the sample with high infection load (replicates 1a–d). The results showed little variability between the two droplet generation events for the same samples with average concentrations of 88.7 copies/µl in cartridge 1 and 86.1 copies/µl in cartridge 2 (Table 3), % coefficient of variation (%CV) being 8.8 and 9.5, respectively (Figure 2). For the sample with intermediate infection load (replicates 2a–d), the mean number of copies/µl was 49.8 (SE =1.2), ranging from 46.6 to 53.0 (Figure 2). The accuracy of the concentration measurements was highest in intermediate infection group, where %CV was 4.3 in cartridge 1 and 5.1 in cartridge 2 (Table 3). The mean number of copies/µl for the sample with low infection load was 1.95 (SE =0.28), being 2.32 copies/µl in cartridge 1 and 1.59 copies/µl in cartridge 2. %CV was 27.7 for low infection sample replicates ran in cartridge 1 and 32.9 in cartridge 2. Overall, no significant differences in the COX1 concentration between the droplet generation events were detected (Wilcoxon rank sum test: *W* = 18, *p* = 1.000), the median shift being 0.27 copies/µl (Figure 2).

**TABLE 3:** Concentration of COX1 (copies/µl) in reproducibility experiment ddPCR reactions (95% Poisson CI), mean concentration in each group and standard error of the mean (SE), % coefficient of variation (%CV) in each group, number of accepted droplets, mean amplitude of positive reactions fluorescence, mean amplitude of negative reactions fluorescence, and copies per partition (*λ*)

Sample	Copies/µl (95% CI)	Mean number of copies (SE)	%CV	Droplets	Mean amplitude of positives	Mean amplitude of negatives	*λ*
High_a	83.2 (78.1–88.4)			14,522	17,024	5604	0.071
High_b	94.2 (88.9–99.6)			15,489	16,819	5569	0.080
High_c	80.3 (75.2–85.5)			14,140	16,703	5190	0.068
High_d	91.9 (86.7–97.1)	87.8 (3.35)	6.6	15,815	16,474	5130	0.078
Intermediate_a	46.6 (42.9–50.3)			15,638	16,599	5284	0.040
Intermediate_b	49.5 (45.7–53.4)			15,379	16,761	5293	0.042
Intermediate_c	53.3 (49.3–57.3)			15,127	16,477	4913	0.045
Intermediate_d	49.6 (45.7–53.5)	49.8 (1.2)	4.8	15,211	16,608	4881	0.042
Low_a	1.86 (1.2–2.74)			14,527	16,867	5348	0.002
Low_b	2.77 (1.92–3.86)			13,584	17,252	5379	0.002
Low_c	1.96 (1.27–2.85)			14,451	16,716	4890	0.002
Low_d	1.22 (0.69–1.98)	1.95 (0.28)	28.2	13,490	17,150	4921	0.001
*S. pygospionis*	0	—		15,015	0	4701	—
*P. pygospionis*	0	—		16,931	0	4354	—
Neg. 1	0	—		16,049	0	4669	—
Neg. 2	0	—		17,322	0	4321	—

Regression analysis showed that the concentration of the 10‐fold dilution series had good linearity (*R* ^2^ > .99, *p* < .001) (Figure 4). The dynamic range of the ddPCR assay reached as low as 1:1000 dilution (mean concentration =0.22, SE =0.03 copies/µl) (Table 4). However, one replicate did not show any amplification; hence, LOD was determined to be 0.86 COX1 copies/µl (1:100 dilution), as all 10 replicates had more than 3 positive droplets (Table 4). %CV was lowest for the undiluted original DNA sample and highest in 1:1000 dilution (Table 4).

**TABLE 4:** Concentration of COX1 (copies/µl) in 10‐fold dilution experiment ddPCR reactions (95% Poisson CI), mean concentration in each group and standard error of the mean (SE), %CV, number of accepted droplets, number of positive droplets, mean number of positive droplets, and number of copies per partition (*λ*)

Sample	Copies/µl (95% CI)	Mean number of copies (SE)	%CV	Droplets	Positive droplets	Mean number of positive droplets (SE)	*λ*
1:1 Rep1*	*74.2 (62.3–86.2)*			*2422*	*148*		*0.063*
1:1 Rep2	88.6 (82.4–95.0)			10,485	761		0.075
1:1 Rep3	97.6 (916–104.0)			12,917	1028		0.083
1:1 Rep4	90.4 (84.0–96.8)			10,440	772		0.077
1:1 Rep5	88.3 (82.1–94.5)			10,874	786		0.075
1:1 Rep6	86.0 (79.9–92.2)			10,762	759		0.073
1:1 Rep7	95.4 (89.4–101.0)			12,396	966		0.081
1:1 Rep8	93.1 (87.1–99.1)			12,302	936		0.079
1:1 Rep9	86.0 (79.9–92.1)			10,811	762		0.073
1:1 Rep10	105.0 (99.0–111.0)	92.27 (1.964)*	6.4*	14,650	1249	891 (297)*	0.089
1:10 Rep1	10.5 (8.6–12.5)			12,695	113		0.009
1:10 Rep2	7.83 (6.3–9.4)			15,226	101		0.007
1:10 Rep3	10.1 (8.3–11.8)			15,248	130		0.009
1:10 Rep4	8.34 (6.9–9.8)			17,553	124		0.007
1:10 Rep5	9.33 (7.7–11.0)			15,321	121		0.008
1:10 Rep6	7.86 (6.4–9.6)			13,965	93		0.007
1:10 Rep7	7.14 (5.6–9.0)			11,404	69		0.006
1:10 Rep8	8.64 (6.9–10.6)			11,477	84		0.007
1:10 Rep9	9.11 (7.4–11.0)			12,831	99		0.008
1:10 Rep10	7.72 (6.0–9.7)	8.66 (0.33)	11.9	10,239	67	100.1 (31.7)	0.007
1:100 Rep1	1.04 (0.52–1.84)			11,272	10		0.0009
1:100 Rep2	0.41 (0.12–0.96)			11,613	4		0.0002
1:100 Rep3	0.41 (0.12–0.97)			11,414	4		0.0004
1:100 Rep4	0.82 (0.39–1.49)			12,886	9		0.0007
1:100 Rep5	1.28 (0.70–2.10)			12,001	13		0.0010
1:100 Rep6	0.71 (0.3–1.39)			11,554	7		0.0006
1:100 Rep7	0.66 (0.28–1.28)			12,556	7		0.0006
1:100 Rep8	0.86 (0.39–1.61)			10,979	8		0.0007
1:100 Rep9	1.45 (0.81–2.35)			11,371	14		0.0012
1:100 Rep10	0.94 (0.47–1.66)	**0.86 (0.10)**	37.5	12,508	10	8.6 (2.7)	0.0008
1:1000 Rep1	0.18 (0.03–0.57)			13,182	2		0.0002
1:1000 Rep2	0.27 (0.06–0.7)			13,343	3		0.0002
1:1000 Rep3	0.09 (0.004–0.44)			12,921	1		0.0001
1:1000 Rep4	0.29 (0.07–0.77)			12,112	3		0.0002
1:1000 Rep5	0.33 (0.10–0.77)			14,343	4		0.0003
1:1000 Rep6	0.0 (0.00–0.32)			11,181	0		0
1:1000 Rep7	0.31 (0.07–0.81)			11,573	3		0.0003
1:1000 Rep8	0.27 (0.066–0.73)			12,788	3		0.0002
1:1000 Rep9	0.18 (0.03–0.59)			12,891	2		0.0002
1:1000 Rep10	0.28 (0.07–0.74)	0.22 (0.03)	45.7	12,678	3	2.4 (0.76)	0.0002
NTC1	0 (0.0–0.28)			12,442	0		—
NTC2	0 (0.0–0.21)			16,933	0		—
NTC3	0 (0.0–0.25)			14,014	0		—

Note

Concentration for limit of detection is shown in bold. *Replicate 1 in the undiluted sample (in italic font) was discarded from the analysis because of low droplet count: hence, group means are calculated only with nine replicates.

**FIGURE 3:**
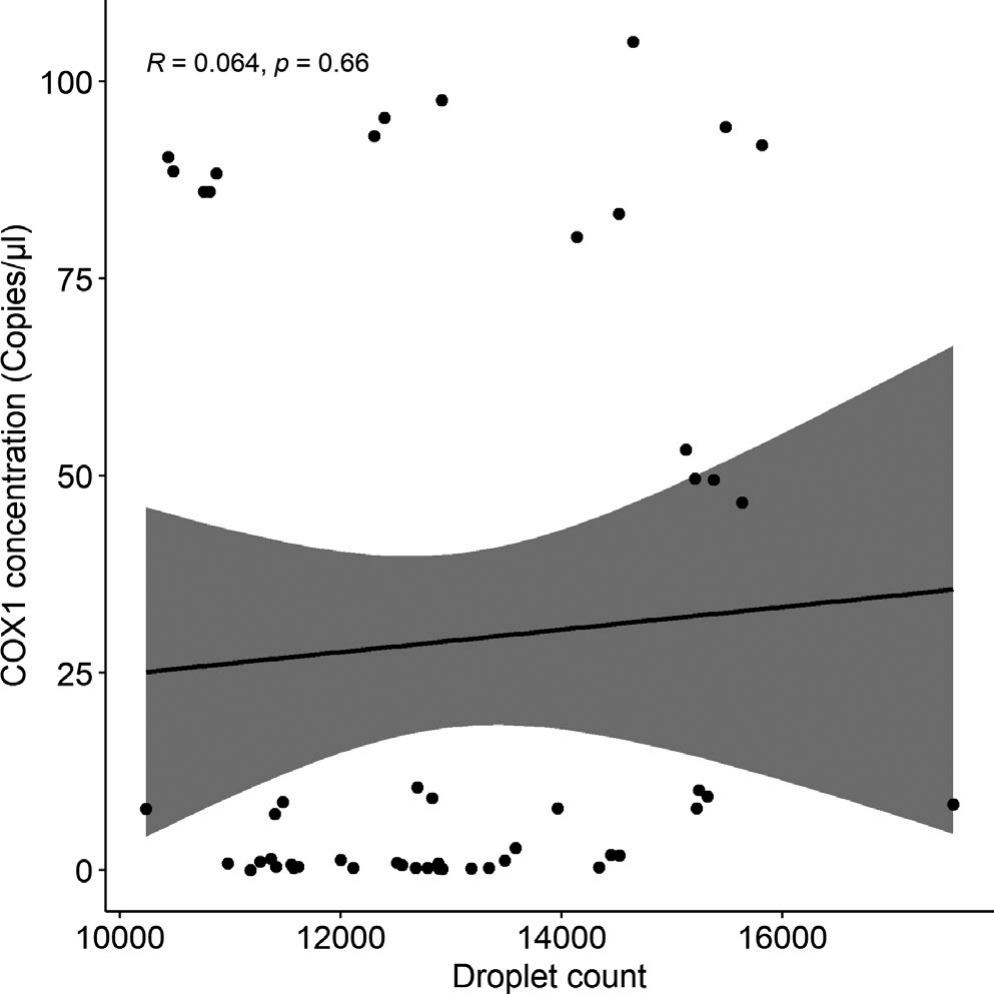
Non‐significant Pearson's correlation between the COX1 concentration (copies/µl) and the droplet count (*n* = 51)

**FIGURE 4:**
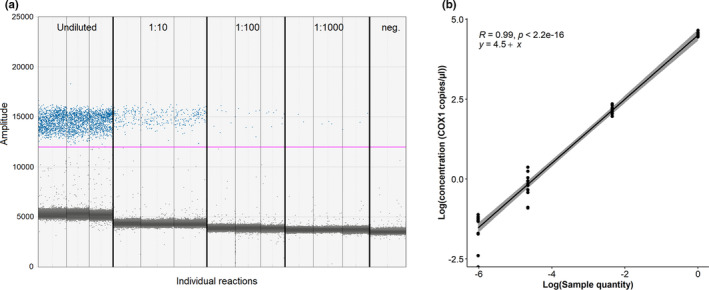
Ten‐fold dilution series to detect the lowest limit for agamococcidian detection for the ddPCR assay. (a) 1D ddPCR plot. *x*‐axis shows an example of individual reactions for the undiluted, 1:10, 1:100, and 1:1000 diluted replicates, and *y*‐axis shows the amplitude of the fluorescence. Positive reactions (blue) and negative reactions (grey) are separated by manually set threshold (pink line, at amplitude 11,309). Negative controls (neg) did not show any amplification above the threshold. (b) Dilution series sample quantity in log scale plotted against COX1 concentration (copies/μl) in log scale in each replicate. Significant correlation coefficient (*r* ^2^ = .99, *p* < .001) between the absolute concentration (COX1 copies/µl) and 10‐fold dilution series shows good linearity of the assay

Absolute concentration measurements were independent of the droplet count (Pearson's correlation: *t* = 0.449, df =49, *p* = .655) (Figure 3). The droplet count varied from 10,239 to 17,553. Positive and negative droplets were easily separated from each other with threshold set to amplitude 13,033, mean amplitude for positives being 16,788, and negatives 5028. Some “rain” between the positive and negative clusters was observed, potentially because of the formation of primer dimer and the presence of background amplification (Figures 2a and 4a). The ddPCR assay did not amplify other apicomplexans (*S. pygospionis* and *P. pygospionis*) possibly present in the total DNA extractions from hosts (concentration 0 copies/µl) (Figure 2a).

### 3.2 Prevalence of infection within host populations

**FIGURE 5:**
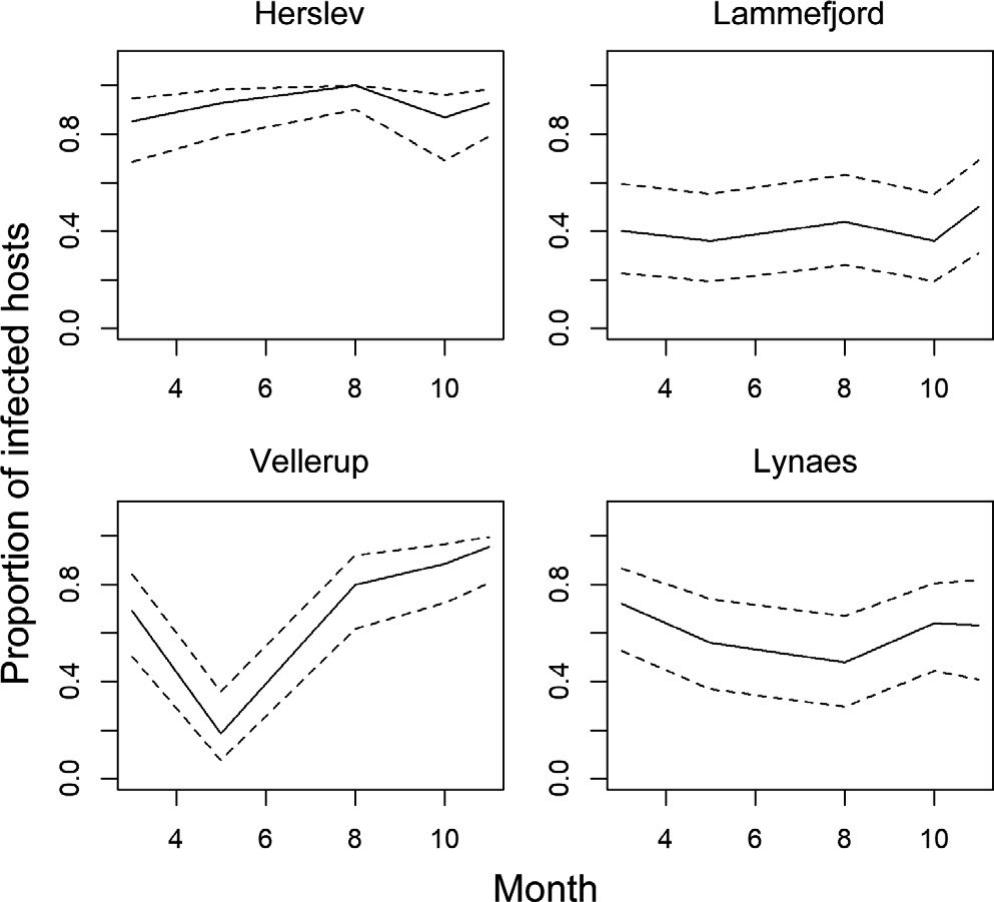
Proportion of infected host individuals through the sampling period in each population. Dashed lines indicate the 95% confidence intervals

Prevalence of infection differed between the populations (*Χ* ^2^ = 64.9, df =3, *p* > .001). The highest proportion of infected hosts ranged from 85% to 100% in Herslev, where it was 94.9% more likely that the worms were infected than in Lammefjord, 77.1% more likely than in Vellerup, and 91.8% than in Lynæs (Table 5). The lowest prevalence was found in Lammefjord, where fewer than 50% of hosts were infected throughout the sampling period (Figure 4, Table 5). No clear seasonal pattern common to the four populations was found (*Χ* ^2^ = 4.3, df =4, *p* = .36). The highest prevalence was observed in Herslev in August (of all studied populations and sampling times) and the lowest in Vellerup in May (Figure 5). Although the probability of being infected increased towards November, the increase was not significant (Table 5).

**TABLE 5:** Pooled logistic regression odds ratios for prevalence of infection

		Odds ratio (95% CI)	SE	*t*	df	*p*‐value
	(Intercept)	6.231 (1.83–21.26)	0.623	2.899	487	<.005
Population	Lammefjord	0.051 (0.02–0.11)	0.381	−7.822	487	<.001
	Vellerup	0.229 (0.11–0.50)	0.393	−3.750	487	<.001
	Lynæs	0.082 (0.04–0.18)	0.400	−6.242	487	<.001
Month	May	1.958 (0.78–4.93)	0.470	1.433	487	.152
	August	1.025 (0.54–1.96)	0.331	0.071	487	.944
	October	1.135 (0.59–2.20)	0.338	0.375	487	.708
	November	1.799 (0.87–3.73)	0.371	1.588	487	.113
Length		1.001 (1.0001–1.001)	0.000	2.199	487	.028
Density		0.999 (0.9994–1.000)	0.000	−3.912	487	<.001

Note

The distribution error function is binomial with a logistic link function. The references for population and month were Herslev and March, respectively. Altogether 499 samples were utilized in this model, including 19–28 individuals per population in each month.

Larger worms had an increased probability to be infected, but the effect was small: For every micrometer increase in length, the probability of being infected was 0.1% higher (Table 5). When host population densities were high, the prevalence of infection was significantly lower, but the effect was small (Table 5). For every host individual increase within a square meter, the probability of infection decreased to 0.04%. Organic content (%) in the sediment was found to decrease the proportion of infected hosts by 67%, but the effect was not significant (*z* = −0.994, df =20.1, *p* = .332), and the predictor was dropped from the final model (likelihood‐ratio test D3: *p* = .335).

We obtained similar results when the analysis was restricted to only complete cases (excluding samples from October). However, logistic regression with multiple imputation was generally more efficient as can be seen from the smaller confidence intervals and lower *p*‐values (Table 6). Also, infection load peaked in Lammefjord and Lynæs in October, which would not have been observed in the complete cases model.

**TABLE 6:** Logistic regression with complete cases only (missing data from October)

		Estimate (95% CI)	SE	*z*‐value	df	*p*‐value
	(Intercept)	1.764 (0.38 to 3.29)	0.718	2.457	397	.014
Population	Lammefjord	−3.014 (−3.94 to −2.20)	0.442	−6.826	397	<.001
	Vellerup	−1.766 (−2.70 to −0.92)	0.449	−3.929	397	<.001
	Lynæs	−2.767 (−3.74 to −1.89)	0.467	−5.918	397	<.001
Month	May	0.708 (−0.23 to 1.68)	0.485	1.458	397	.145
	August	0.020 (−0.63 to 0.67)	0.332	0.060	397	.952
	November	0.601 (−0.13 to 1.35)	0.376	1.597	397	.110
Length		0.001 (0.000 to 0.001)	0.000	2.303	397	.021
Density		−0.0003 (−0.001 to 0.000)	0.000	−3.790	397	<.001

Note

The distribution error function is binomial with a logistic link function. The references for population and month are Herslev and March, respectively. Altogether 398 samples were utilized in this model, including 19–28 individuals per population in each month.

### 3.3 Infection load in natural host populations

The largest range of agamococcidian COX1 gene copies detected by ddPCR was in the Herslev population, ranging between 0.86 and 1700.98 copies/µl. In Lammefjord, the range of detection was from 0.86 to 53.70 copies/µl, in Vellerup from 0.86 to 807.62 copies/µl, and in Lynæs from 0.86 to 665.30 copies/µl.

The agamococcidian infection was highly aggregated in most populations and throughout the sampling period (Table 7.) Variance to mean ratio was high in all populations except in Lammefjord in May and in August, when also the mean infection load was lowest (from 0.4 to 1.62 COX1 copies/ng of total DNA). The infection was most aggregated in October (Lammefjord and Lynæs) and in November (Herslev and Vellerup).

**TABLE 7:** The prevalence of infection (%) and number of infected hosts (*n*), mean infection load (COX1 copies/ng of total DNA) ($\bar{x}$), the standard error for the mean (SE), variance (*s* ^2^), and variance to mean ratio (*s* ^2^/$\bar{x}$) in the studied populations

		% (*n*)	$\bar{x}$	SE	*s* ^2^	*s* ^2^/$\bar{x}$
March	Herslev	85.2 (23)	101.10	46.10	48,915.70	483.83
	Lammefjord	40.0 (10)	0.40	0.10	0.10	0.30
	Vellerup	69.2 (18)	1.30	0.50	3.70	2.80
	Lynaes	72.0 (18)	18.20	13.60	3333.00	183.10
May	Herslev	92.9 (26)	104.90	43.50	49,265.50	469.60
	Lammefjord	36.0 (9)	1.62	0.50	2.16	1.30
	Vellerup	18.5 (5)	59.70	58.20	16,937.52	283.71
	Lynaes	56.0 (14)	1.10	0.30	1.50	1.38
August	Herslev	100.0 (24)	150.45	64.30	99,181.60	659.20
	Lammefjord	44.0 (11)	0.75	0.20	0.44	0.59
	Vellerup	80.0 (20)	351.60	126.00	317,762.80	903.76
	Lynaes	48.0 (12)	2.61	1.41	24.01	17.03
October	Herslev	87.0 (20)	210.20	106.60	227,294.40	1081.30
	Lammefjord	36.0 (9)	72.20	65.20	38,222.87	529.40
	Vellerup	88.5 (23)	140.40	8.40	14,8597.40	1058.39
	Lynaes	64.0 (16)	250.40	197.60	62,4783.70	2495.14
November	Herslev	92.9 (26)	349.20	145.20	548,069.20	1569.50
	Lammefjord	50.0 (12)	24.40	23.90	6826.50	279.78
	Vellerup	95.5 (21)	164.50	10.10	227,784.40	1384.71
	Lynaes	63.2 (12)	19.00	15.60	2933.20	154.38

**FIGURE 6:**
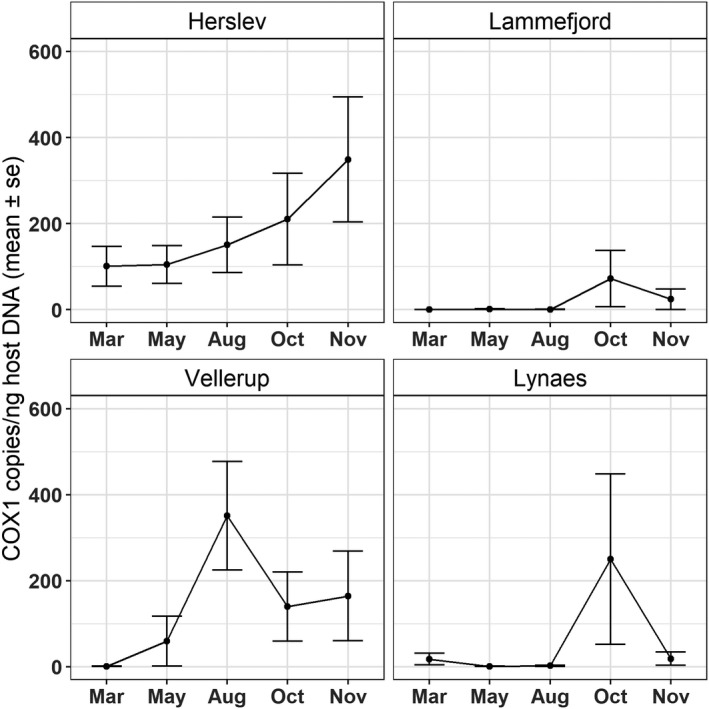
Mean infection load (COX1 copies/ng total DNA) in the four populations over the sampling period. Error bars indicate the standard error of the mean

Linear regression analysis (*r* ^2^ = .272) showed that the infection load differed between populations (*F* = 29.30, df =3, *p* > .001). In Herslev, the infection load was high throughout the sampling season, ranging from 101.10 to 349.20 copies/ng total DNA. The lowest infection load was found in Lammefjord, where the range was from 0.40 to 72.20 copies/ng total DNA. The infection load changed seasonally (*F* = 8.08, df =4, *p* > .001) being highest in October (Figure 6, Table 8). Overall, the highest mean infection load was found in Vellerup ($\bar{x}$ = 351.6 copies/ng of total DNA, SE =126) in August, and the lowest in Lammefjord in March ($\bar{x}$ = 0.4 copies/ng of total DNA, SE =0.1) (Table 7, Figure 6).

**TABLE 8:** Linear regression coefficients for the log‐transformed infection load

		Estimate (95% CI)	SE	*t*‐statistic	df	*p*‐value
	(Intercept)	1.70 (1.05 to 2.35)	0.330	3.630	319	<.001
Population	Lammefjord	−3.16 (−3.92 to −2.41)	0.386	−8.20	319	<.001
	Vellerup	−0.99 (−1.38 to −0.32)	0.330	−2.99	319	<.005
	Lynæs	−2.28 (−2.96 to −1.61)	0.345	−6.61	319	<.001
Month	May	0.93 (0.10 to 1.76)	0.422	2.19	319	.029
	August	1.53 (0.75 to 2.31)	0.396	3.86	319	<.001
	October	1.93 (1.16 to 2.71)	0.394	4.90	319	<.001
	November	1.82 (1.05 to 2.59)	0.391	4.65	319	<.001

Note

The references for population and month were Herslev and March, respectively. The distribution error function is normal. Because only infected hosts were used in the analysis, the sample size was 329 in total, including 5–26 individuals per population in each month. Analysis was performed without multiple imputation since the variables with missing data (length and organic content %) were not significant.

Higher organic content in the sediment was associated with lower infection load, but the effect was not significant (*t* = −1.579, df =140, *p* = .117). Also, the length of the host did not have a significant effect on the infection load (*t* = −0.495, df =315, *p* = .621), nor did the host population density (*t* = 0.806, df =316, *p* = .421), and these variables were left out of the final model, which was performed for the original, non‐pooled data without multiple imputation.

## 4 DISCUSSION

In this study, we show that ddPCR can be used as a reliable tool to quantify symbionts from whole host DNA extracts. We developed a ddPCR assay to detect and quantify an undescribed agamococcidian based on its COX1 gene, and we demonstrated its use in studying infection dynamics by documenting the prevalence of the symbiont and the infection load in four seasonally sampled populations of its host, *P. elegans*. ddPCR can enable further investigation of cryptic symbiotic interactions that are difficult to carry out with other molecular methods and impossible with morphological methods alone. Here, the agamococcidian COX1 was detected in amounts ranging from 0.04 to >3000 copies per ng of total DNA extracted from hosts (mixtures of host and symbiont DNA).

As expected for ddPCR, our assay was reproducible and precise, similar to ddPCR assays used for symbiont quantification in previous studies (Koepfli et al., 2016; Srisutham et al., 2017; Wilson et al., 2015). Results were not affected by droplet generation event (Figure 2), and variation in the concentration was small especially when the number of copies/µl was high (Figures 2 and 3). The precision of ddPCR is expected to increase with an increasing number of partitions (Quan et al., 2018). In our experiments, the droplet count varied from 10,239 to 17,553, which is typical for droplet‐based methods, and it did not correlate with target concentration (Figure 3). Additionally, the precision is affected by the average number of target molecules per droplet (*λ*), since an uneven distribution of template across the partitions might cause a decrease in precision for very low target concentration samples (Hindson et al., 2013; Strain et al., 2013). Our experiments showed an even distribution of targets per droplet; however, low *λ* and higher %CV values were detected for very low target concentrations (Tables 3 and 4).

As ddPCR results in absolute concentration and no standard curves are needed (Hindson et al., 2013), the method is particularly applicable for quantifying undescribed symbionts from total DNA extracts of hosts, when extraction of the symbionts is not feasible to provide the reference material needed when preparing standard curves (Salipante & Jerome, 2019). Importantly, our assay can detect symbionts at a very low density (0.86 copies/µl), which might realistically be expected for agamococcidians and other apicomplexans. Calculation of infection loads for agamococcidians has not been attempted previously, but Miroliubova et al. (2020) remarked that they found from a few up to several hundreds of individual rhytidocystids within the polychaete *Ophelia limacine*. Moreover, the assay developed here did not amplify the other apicomplexan symbionts (*S. pygospionis* and *P. pygospionis*) that might also be present within the hosts, showing the specificity of the designed primers. Hence, the ddPCR assay not only enables monitoring of symbionts that are otherwise difficult to quantify but also makes it possible to study co‐infections of multiple apicomplexan species across host populations efficiently in terms of both time and labor (A.‐L. Hiillos et al., in prep).

However, because our assay is based on the mitochondrial COX1 gene, the quantification of gene copies given by the ddPCR assay does not indicate the exact number of symbiont cells that are present in a single host individual or how many individuals is the minimal level of detection. A single symbiont cell can potentially harbor multiple copies of the mitochondrial marker. For example, in *S. pygospionis*, the number of mitochondria increases with cell size (Paskerova et al., 2018). To achieve more accurate estimates of the symbiont count, a single copy nuclear marker would be better suited for the assay. Considering how well the target symbiont is known and whether genetic data from the symbiont is available, it could be difficult to design primers with sufficient specificity to use with total host DNA extracts because of conservation of gene sequences shared with the host or other related symbiont species. We used the COXI gene fragment because it was available and sufficiently different from the host COXI sequence (Supporting information 1) and because the COXI gene has shown potential for DNA barcoding of some protists (Pawlowski et al., 2012) including apicomplexans (Ogedengbe et al., 2011). It would be interesting to follow up our study using a different assay that could provide more exact counts. Regardless, our ddPCR assay based on COXI not only allows for detection of the undescribed agamococcidian but also provides an indication of the symbiont infection load, allowing for further insight into symbiont dynamics and biological interactions between symbiont and host.

Use of our ddPCR assay to survey natural populations of the host revealed a dynamic pattern of infection by the agamococcidian in the studied host populations. The proportion of infected *P. elegans* was high overall (85.7%), but it differed between the populations, and it remained constant throughout the sampling season in most populations. The highest prevalence was found in Herslev, where almost all hosts were infected. Prevalence was low in Lammefjord, ranging from 36% to 50%, and intermediate in Lynaes, where it ranged from 48% to 72%. Only in Vellerup, prevalence changed over the season, from high (69.2%) in March to very low in May (18.5%), and increasing again towards fall so that 95% of hosts were infected in November. Similarly, the highest mean infection load was found in Herslev and the lowest in Lammefjord. Infection load changed seasonally increasing towards fall: peaking earliest in August in Vellerup, but otherwise highest in October (Lammefjord and Lynaes) and November (Herslev). Furthermore, infection load was highly aggregated in all populations, meaning that few individuals in the population harbor majority of the symbionts. The symbiont distribution within infected hosts also showed seasonal dynamics, with low or almost even variance to mean ratios in some populations in spring and summer but more aggregation in the fall. Although apicomplexan dynamics in marine environments have not been extensively studied, seasonal changes in prevalence and/or infection intensity have been observed in some host–symbiont systems. For example, apicomplexan infection in the Iceland scallop showed high prevalence throughout the seasons, with infection load being highest in spring (Kristmundsson et al., 2015). In another study, prevalence of apicomplexans infecting reef‐building corals was low during summer months and increased towards fall (Kirk et al., 2013). In contrast, some marine apicomplexan infections are found to remain stable throughout seasons (Halliday‐Isaac et al., 2021), suggesting that the infection dynamics of marine apicomplexans are case specific and require more thorough examination.

Apicomplexans are generally expected to be parasitic (Morrison, 2009), but how the agamococcidian studied here affects the host's fitness is not known. Aggregated distribution within hosts is typical for parasitic symbionts (Anderson & May, 1978), and we expect that the same factors influencing parasite dynamics on a general level, specifically encounter rates and host susceptibility (Schmid‐Hempel, 2011), could also be relevant for the studied species. Marine apicomplexans are thought to have simple life cycles with one host species and passive oral–fecal transmission among hosts (e.g., Leander, 2007). Therefore, high host densities are expected to increase prevalence and infection loads (Anderson & May, 1978; Arneberg et al., 1998) since symbiont transmission is more efficient because of increased encounter rates (Patterson & Ruckstuhl, 2013; Rifkin et al., 2012). However, we found that when host density was highest (in May), prevalence of infection did not increase accordingly. In addition, the infection load was lower when host density was high, although the estimate was not significant. A similar non‐significant negative effect of host density on prevalence was detected in apicomplexans infecting amphipods (Grunberg & Sukhdeo, 2017). Those results, together with ours, suggest that the relationship between host density and prevalence or infection load is not necessarily straightforward and is possibly modified by other characteristics of both host and symbiont biology.

For instance, seasonality in host reproduction and reproduction strategy might affect prevalence and infection loads (Šimková et al., 2005; White et al., 1996) via changes in encounter rates and susceptibility. The studied host populations show seasonal dynamics in their reproduction, with reproductive peaks occurring in both spring and fall (Thonig et al., 2016). We observed that infection load and symbiont aggregation were high in October and November when the proportion of reproducing hosts also increases, perhaps because of an increase in food consumption to enable gamete production and consequential exposure to agamococcidian oocysts. However, we did not observe a similar peak in spring. Additionally, the host populations differ in the main mode for larval development (Thonig et al., 2016). Prevalence and infection loads were highest throughout the sampling season in Herslev, where the worms are known to produce mainly large, non‐dispersing benthic larvae. Interestingly, low prevalence and infection loads were detected in Lammefjord and Lynæs, where the main developmental mode is via planktonic larvae, which are capable of dispersing. Because of these developmental differences, a more constant supply of susceptible hosts might be present in Herslev compared with Lammefjord and Lynæs, where influx of new cohorts via planktonic larvae might be less predictable. The pattern and association of infection with developmental mode demand additional study.

Another host characteristic that affects symbiont encounter is host body size (e.g., Taskinen & Valtonen, 1995). Larger size is generally connected to older age or increased consumption rates, which could further increase encounter rates with the symbiont. We found that larger worms were more likely to be infected, but host size did not affect the infection load. In a system where two apicomplexans are infecting the same host species, Grunberg and Sukhdeo (2017) found that larger and older hosts had higher infection load of one apicomplexan species but not the other, leading them to propose that changes in host feeding patterns along with seasonal changes in host demographics might drive changes in symbiont abundance. The host species in our study, *P. elegans*, can both filter feed and deposit feed (Anger et al., 1986), deposit feeding being more common in larger worms (Hentschel, 1998), which could explain our observation of larger worms being more likely to be infected. As the available food type can affect the grazing behavior in polychaetes (Jordana et al., 2001), filter feeding in *P. elegans* might be more common in spring when phytoplankton is more abundant. A change in feeding behavior with season might reflect the infection load patterns observed in this study, since hosts are more likely to encounter symbiont infective stages while deposit feeding. This could also explain why we did not observe an increase in parasite load during the spring reproductive peak. Even though reproducing hosts have higher consumption rates to support developing gametes, if the increased consumption is through filter feeding in spring, agamococcidian oocysts could be avoided. Furthermore, the symbiont's life history might also affect the encounter rates. Many marine symbionts accelerate their reproduction rate inside their hosts during warm seasons (Overstreet & Lotz, 2016). Consequently, there could be a progressive accumulation of oocysts in the sediment towards fall. Since apicomplexan oocysts are known to be very persistent (Clopton et al., 2016), oocysts produced in summer can remain viable until fall when hosts encounter rate possibly is higher because of the afore‐mentioned features in host biology.

We observed differences among host populations and did not find a common seasonal pattern of infection, suggesting that environmental factors unrelated to broad seasonal changes could be influencing symbiont and host interactions. In this study, the environmental factors are all represented by the organic content % in the sediment, since other variables measured (salinity, temperature, and sediment characteristics) were correlated with host density. We chose to retain organic content in our analysis since it measures the amount of nutrients available for the host to use (e.g., Cheng et al., 1993). Higher organic content was found to lower infection load and the prevalence of infection, even though the effect was not statistically significant. Low organic content in sediment has been correlated with larger foraging area in the polychaete *Streblospio benedicti* (Kihslinger & Woodin, 2000) and could therefore increase the encounter rate of the host with symbiont oocysts and lead to heavier infection. The pattern could also indicate that hosts living in a habitat with higher organic content could be in a better condition and thus have better immunity against the infection. However, as the nature of the interaction between the symbiont and its host (parasitic or mutualistic) and possible immunity is not currently known, further studies are needed. In addition, it is important to remember that one of the other environmental variables correlated with organic content % might be more relevant biologically for explaining the infection patterns, and other unmeasured environmental variables cannot be discounted.

## 5 CONCLUSION

We have shown that ddPCR can be used effectively to detect symbionts and quantify infections from DNA extracts of hosts despite challenges of working with cryptic and undescribed symbiont species. Our assay reliably targets an agamococcidian symbiont in a marine polychaete, *P. elegans*, and provides specific quantification of infection load despite the fact that DNA pools could also contain DNA from other, related apicomplexan species as well as host DNA. We surveyed four natural host populations sampled seasonally and found that infection was population specific and dynamic. A common seasonal pattern of prevalence was not detected. Since very few studies have been able to survey agamococcidians or other marine apicomplexans, our results suggest a concrete avenue towards better understanding of the interaction of marine apicomplexans and their hosts. Effective and efficient tools, such as the ddPCR assay introduced in this study, are required for monitoring the host–symbiont dynamics and the consequences of their biological interactions.

## CONFLICT OF INTEREST

Authors declare no conflict of interest.

## AUTHOR CONTRIBUTIONS

**Anna‐Lotta Hiillos:** Conceptualization (equal); Data curation (lead); Formal analysis (lead); Funding acquisition (equal); Investigation (equal); Methodology (equal); Project administration (equal); Validation (equal); Visualization (lead); Writing‐original draft (lead); Writing‐review & editing (equal). **Anne Thonig:** Data curation (equal); Investigation (equal); Methodology (equal); Resources (equal); Writing‐original draft (supporting); Writing‐review & editing (equal). **Karelyn Emily Knott:** Conceptualization (equal); Data curation (equal); Formal analysis (equal); Investigation (equal); Methodology (lead); Project administration (equal); Resources (equal); Supervision (lead); Validation (equal); Writing‐original draft (equal); Writing‐review & editing (equal).

## Supporting information

Supplementary Material

## Data Availability

All data and R scripts are uploaded to Dryad repository (https://doi.org/10.5061/dryad.jwstqjq90). Sequence data generated in this study from two isolates of agamococcidian COX1 with accession numbers OK562425 and OK562426 will be available in NCBI GenBank upon acceptance.
