# Supplementary material for: Droplet digital PCR as a tool for investigating dynamics of cryptic symbionts
Source: Ecol Evol. 2021 Nov 22;11(23):17381–96. doi: 10.1002/ece3.8372 (PMC8668802; doi:10.1002/ece3.8372)
Supplement: Supplementary file 1 — Supplementary Material [file ECE3-11-17381-s001.docx]

**Droplet digital PCR (ddPCR) as a tool for investigating dynamics of cryptic symbionts**

Anna-Lotta Hiillos, Anne Thonig & K. Emily Knott

**Supplement 1: Sequence Analyses**.

To ensure the that the designed primers amplified the expected product, we used standard DNA sequencing methods to investigate amplified products from infected host polychaetes. Sequencing protocols follow those described in Kesäniemi et al. 2012, except the primers used were the ApiCox1F and ApiCox1R primers described in this paper with amplification annealing temperature set to 58 ^o^C. Samples of the host *Pygospio elegans* had been used in the Kesäniemi et al. 2012 study and came from populations from Finland, Denmark, Scotland and Iceland. DNA extracts of the host worms made in the previous study had been stored at -20 ^o^C. All sequencing steps were done in our laboratory.

The fragment amplified by the ApiCox1F and ApiCox1R primers is 226 bases long (including primer sequences). The sequences obtained were essentially identical among all tested samples (n = 15) and only one variable position was found. The sequenced fragment was identified as a part of the cytochrome oxidase c subunit I gene (COXI) using BLASTx and BLASTn searches to databases maintained by the National Center for Biotechnology Information (<https://blast.ncbi.nlm.nih.gov/Blast.cgi> ; 21 May 2021).

>ApiCOX1 amplicon

ACTGGTCTATCAAGTGTACTGGCTTCAGTAAACTTCCTATCAACAATTGCATGTATTAAAGCAACAGGTAAGTTACTAGGTCAAATGTCTTATTTCGTATGGGGAATA/CATTTTTACTGCCATCTTATTAGTTTTAACTATACCTATTTTAAGTAGTGGTCTACTAATGCTATTAGCTGATTATACATACTGTACTATATTCTATGACCCTGAATTTAGTGGTGATC

A BLASTx search of the nonredundant protein database using an expect threshold of 0.001 and a limit of 10 hits yielded matches to sequences of Apicomplexa (including *Haemogregarina* sp., e.g Accession QSC88272; *Parahaemoproteus* sp. DR05_DR90 Accession ADK75026; several *Haemoproteus* species, e.g. AIY32630.1; and *Plasmodium* sp. ALE28179.1), All hits had 98% coverage and similar scores (80.1 - 86.3), E-values (10^-17^- 10^-19^) and % identity (47.30 - 54.05%). BLASTn searches to the non-redundant nucleotide database yielded hits to various species (fungi, Coleoptera, and the apicomplexan *Leucocytozoon* sp. MAP-2019b), but with low query coverage (< 55%) and alignment scores.

Because new transcriptome datasets for poorly represented Apicomplexa are now available in the database (see e.g. Janouškovec et al. 2019), we searched for matches among these sequences, by limiting BLASTn searches to the Transcriptome Shotgun Assembly (TSA) database limiting searches to sequences of Apicomplexa (taxid:5794). In these searches, 100 best hits with a minimal expect threshold of 0.001 were allowed. The result of these searches indicated that our amplified sequence had the best match to sequences from *Rhytidocystis* sp. ex. *Travisia forbesii*.isolate WS-2016 (Accession: GHVS01023304.1) with query coverage 99% and identity 71.56%. There were no significant matches to other Apicomplexa in the TSA database.

Building upon the sequenced fragment, we designed new primers, clarified the full-length sequence of the COX1 gene. In BLASTx searches to the non-redundant protein database, all matches were to Apicomplexa, with the best match to cytochrome c oxidase subunit I of Apicomplexa sp. WK-2018_Corallicola (Accession: AXQ37355) (Figure 2)


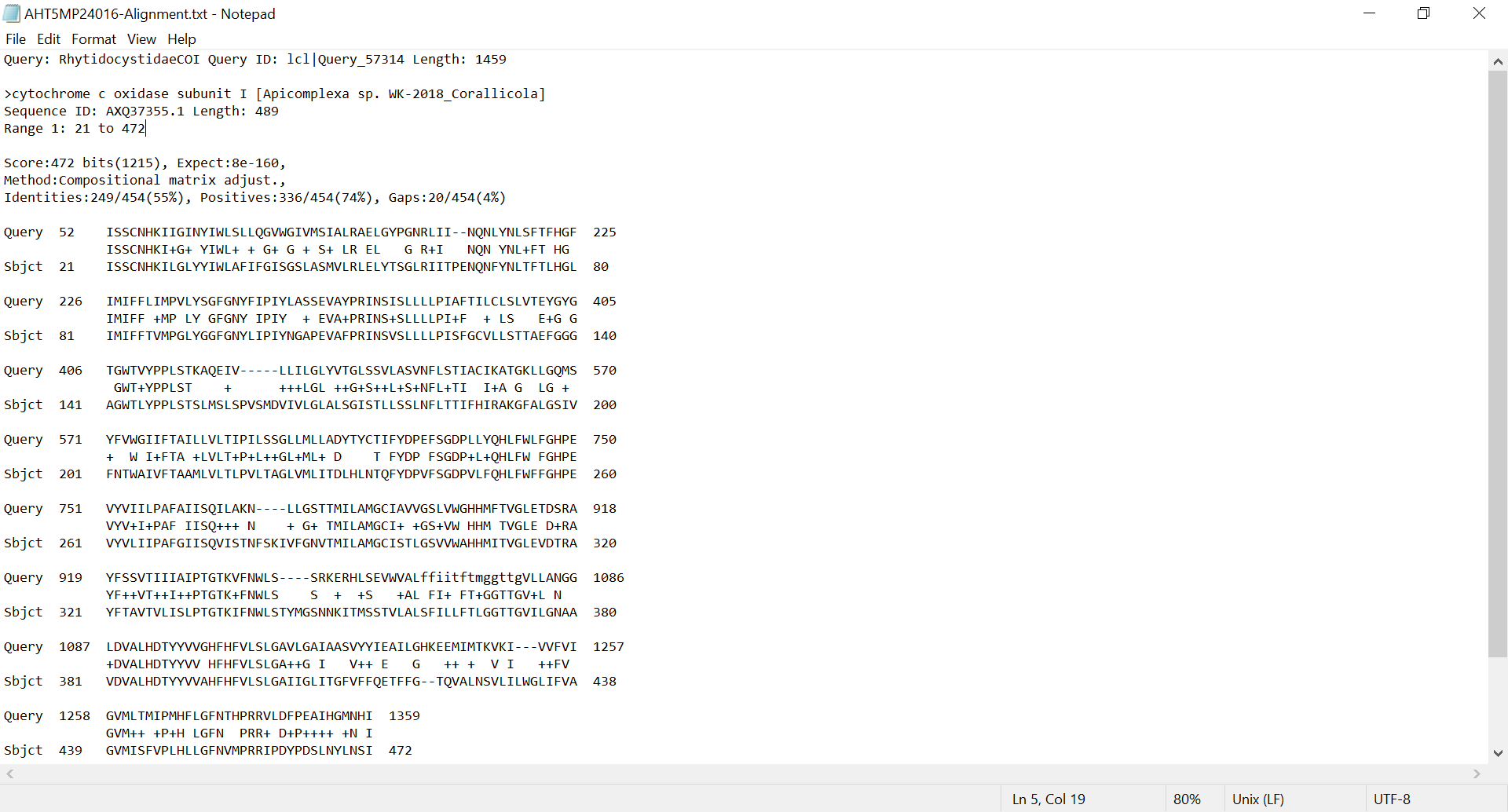


Reciprocal tBLASTn searches of the Transcriptome Shotgun Assembly (TSA) database as above, but using the full-length translation of our obtained sequence as query, yielded best matches to sequences from *Rhytidocystis* sp. ex. *Travisia forbesii* isolate WS-2016 (e.g. Accession: GHVS01023299.1) with query coverage 97% and identity 60.17% and the lowest e value possible. Second-best matches were to *Eleutheroschizon duboscqi* isolate WS-2016, Accession GHVT01048480.1.

As a result of these comparisons, we are confident that our ddPCR assay targets the cytochrome oxidase c subunit I gene to an undescribed species in the Rhytidocystidae (Apicomplexa).

References:

Janouškovec, J., Paskerova, G.G., Miroliubova, T.S., Mikhailov, K.V., Birley, T., Aleoshin, V.V., & Simdyanov, T.G. (2019). Apicomplexan-like parasites are polyphyletic and widely but selectively dependent on cryptic plastid organelles. *eLife*, 8:e49662 doi.org/10.7554/eLife.49662.001.

Kesäniemi, J. E., Rawson, P., Lindsay, S., & Knott, E. (2012). Phylogenetic analysis of cryptic speciation in the polychaete *Pygospio elegans*. *Ecology and Evolution*, 2(5):994-1007. https://doi.org/10.1002/ece3.226
